# Advancing global change biology through experimental manipulations: Where have we been and where might we go?

**DOI:** 10.1111/gcb.14894

**Published:** 2019-11-29

**Authors:** Paul J. Hanson, Anthony P. Walker

**Affiliations:** ^1^ Environmental Sciences Division and Climate Change Science Institute Oak Ridge National Laboratory Oak Ridge TN USA

**Keywords:** elevated CO_2_, environment, experiments, models as hypotheses, nutrients, ozone, temperature, warming, water availability

## Abstract

This commentary summarizes the publication history of *Global Change Biology* for works on experimental manipulations over the past 25 years and highlights a number of key publications. The retrospective summary is then followed by some thoughts on the future of experimental work as it relates to mechanistic understanding and methodological needs. Experiments for elevated CO_2_ atmospheres and anticipated warming scenarios which take us beyond historical analogs are suggested as future priorities. Disturbance is also highlighted as a key agent of global change. Because experiments are demanding of both personnel effort and limited fiscal resources, the allocation of experimental investments across Earth's biomes should be done in ecosystems of key importance. Uncertainty analysis and broad community consultation should be used to identify research questions and target biomes that will yield substantial gains in predictive confidence and societal relevance. A full range of methodological approaches covering small to large spatial scales will continue to be justified as a source of mechanistic understanding. Nevertheless, experiments operating at larger spatial scales encompassing organismal, edaphic, and environmental diversity of target ecosystems are favored, as they allow for the assessment of long‐term biogeochemical feedbacks enabling a full range of questions to be addressed. Such studies must also include adequate investment in measurements of key interacting variables (e.g., water and nutrient availability and budgets) to enable mechanistic understanding of responses and to interpret context dependency. Integration of ecosystem‐scale manipulations with focused process‐based manipulations, networks, and large‐scale observations will aid more complete understanding of ecosystem responses, context dependence, and the extrapolation of results. From the outset, these studies must be informed by and integrated with ecosystem models that provide quantitative predictions from their embedded mechanistic hypotheses. A true two‐way interaction between experiments and models will simultaneously increase the rate and robustness of Global Change research.

## Graphical abstract

A commentary summarizes the publication history of *Global Change Biology* for works on experimental manipulations over the past 25 years and highlights a number of key publications. A discussion of the future of experimental work is also provided as it relates to mechanistic understanding and methodological needs. Integration of ecosystem‐scale manipulations with focused process‐based manipulations, networks, and large‐scale observations will aid more complete understanding of ecosystem responses, context dependence, and the extrapolation of results related to global change. A true two‐way interaction between experiments and models will simultaneously increase the rate and robustness of such research.

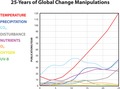

## 1 PUBLICATION TRENDS IN EXPERIMENTAL MANIPULATIONS OVER 25 YEARS OF *GCB*

As a part of this 25th anniversary edition of *Global Change Biology* (*GCB*), a search of the *Web of Science* “All‐Databases” collection for *GCB* publications over its 25 years in business (nearly 5,400 articles) yielded 19.5% that were interpreted to be studies of direct experimental manipulations, field observations across temporal or spatial environmental gradients (that might be interpreted as experiments), or model simulations to address how model‐based hypotheses predict ecosystem responses to climate change experiments. These *GCB* publications on “experiments” produced over 63,000 citations to give an average per publication rate of just under 60 citations per publication.

**Figure 1:**
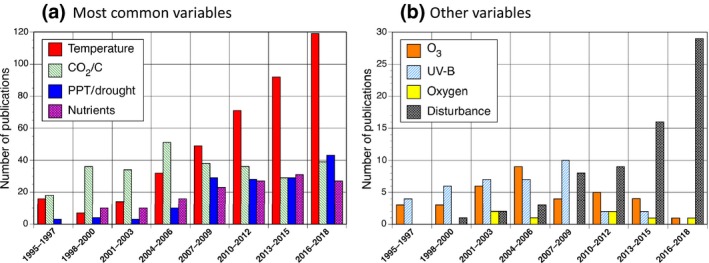
The number of *Global Change Biology* publications on experimental work binned by 3 year intervals and grouped according to the four dominant environmental drivers for the experimental work (a) or the less common drivers (b)

The dominant manipulated environmental variables in *GCB* publications on experiments are *temperature*, *atmospheric CO_2_*, *precipitation or drought*, and *nutrients* (either from atmospheric deposition or fertilization manipulations). Other less commonly manipulated variables include *tropospheric ozone*, *UV‐B radiation*, *disturbance agents*, and a few studies manipulating *oxygen*. The disturbance category included publications on *land use change*, *fire*, wind *storms*, and a variety of unique publications on, for example, *lightning* or *light pollution*. While these *GCB*‐published works were primarily terrestrial vegetation studies, 11.3% of the publications studied aquatic systems (freshwater, ocean margins, or oceans), and 9.7% studied animals. Figure 1 shows publication trends in the type of experimental work over time. At the inception of *GCB*, studies of elevated CO_2_ were most numerous, peaking in the mid‐2000s but with a fairly stable publication rate over the lifetime of *GCB*. In contrast, temperature experiments have continued to increase from a low in the late 90s to by far the most published on manipulation since around 2010. Studies that manipulate aspects of disturbance have also increased dramatically in recent years. Also increasing through time are studies of precipitation change and drought, nutrients. Publications describing the influence of tropospheric ozone and UV‐B were common during *GCB*'s early years but have declined in recent years reflecting a change in funding priorities.

While the authors see evidence that these trends may be somewhat general across the literature, we acknowledge that this assessment of GCB publication trends through time should not be interpreted as a true global trend in experimental effort. The composition of editorial boards and their policies change with time and may thus have an impact on the type and number of publications published within a single journal.

Publications reporting on single‐factor manipulations were initially the most common, but publications evaluating either two‐ or three‐way interactions increased steadily throughout the 25 year history of *GCB* from just two publications per year in 1995 and 1996 to as many as 19 publications per year in the last 5 years. The most commonly studied two‐way interactions included CO_2_ by nutrients (×37 publications) or CO_2_ by temperature (×32 publications) studies. At an extreme, Hanson, Wullschleger, Norby, Tschaplinski, and Gunderson (2005) used a numerical modeling approach to interpolate numerous experimental results to evaluate the relative consequences for known experimental impacts for four different co‐occurring environmental variables: temperature, precipitation change, elevated CO_2_, and tropospheric ozone in eastern deciduous forests.

Meta‐analyses as a statistical means of generalizing results from individual experimental studies (Ainsworth, Rosenberg, & Wang, 2007; Curtis, 1996) have also been well represented and highly cited in *GCB* (Ainsworth, 2008; Ainsworth et al., 2002; De Graaff, Van Groenigen, Six, Hungate, & Van Kessel, 2006; Wu, Dijkstra, Koch, Peñuelas, & Hungate, 2011). However, in this summary, we focus on studies based on reports of primary data.

Publications on experimental or measurement methodologies are not common in *GCB* (only 3% of the experimental publications), but they often develop into influential publications setting the stage for range of influential studies. For example, Granier, Biron, Breda, Pontailer, and Saugier (1996) is a now classic discourse on the use of sap flow methods to assess tree transpiration, and publications by Hollister and Webber (2000), Kimball et al. (2008), and Norby et al. (1997) have all set standards for describing warming technologies important for in situ field manipulation. Such publications are not necessarily the most noteworthy at the time of initial publication, but they become important contributions to experimental science as they are often the primary location of detailed analyses of confounding artifacts associated with a new method. Without such publications, these important caveats may be forgotten with time.

## 2 *GCB* PUBLICATIONS THAT HAVE ADVANCED GLOBAL CHANGE SCIENCE WITH EXPERIMENTS

It was immediately obvious that it would be impossible to do justice to the depth and breadth of the science published in *GCB* over a quarter century, and we have not tried. Instead, a subset of high impact *GCB* publications (i.e., from a list with greater than 100 citations) is summarized as examples of key representative works that may serve as a foundation for informing the next generation of experimental manipulations. Examples are included from *GCB* for publications on the major environmental variables subject to manipulation, publications to elucidate key processes, studies representing results from long‐term observations and networks, and publications on model‐experiment synthesis. Selected publications from other journals published over the last decade are also highlighted to help describe progress in a key research area or support our opinion. This brief list is inexhaustive and based on the personal choice of the authors. Other works and more recent *GCB* publications that are not highlighted here will no doubt rise up and we look forward to seeing that process develops through time.

### 2.1 Manipulations for novel environments

In this section, we highlight a number of publications that describe results from experiments that have manipulated environmental conditions to investigate system‐level responses to the environmental change. The primary goal of these studies is commonly to test systems‐level hypotheses in conditions that minimize experimental artifacts that are often associated with smaller scale manipulations (e.g., free‐air CO_2_ enrichment vs. greenhouse‐based pot studies Hendrey, Ellsworth, Lewin, & Nagy, 1999). It is worth noting that these manipulations are often not intended to mimic a specific future environmental state, rather the manipulation is intended to evaluate system's responses and the processes that govern the systems response to a novel (usually mean) state of a given environmental driver, or few drivers. While process understanding is a goal of these studies, the primary aim is to study responses to a given environmental change.

#### 2.1.1 Elevated carbon dioxide

Leakey, Bernacchi, Dohleman, Ort, and Long (2004) tested the previously suggested insensitivity of C4 *Zea mays* (maize or corn) to free‐air CO_2_ enrichment under favorable growing conditions (i.e., adequate moisture and nutrients). They found, opposite to hypothesis‐based predictions, that growth and net photosynthesis were indeed enhanced when grown under elevated CO_2_. Such a conclusion was made possible through structured experiments where potentially confounding variables were either held constant or adequately quantified to ensure that their impacts would be minimal. Although there are many, a few notable publications summarizing the state of elevated CO_2_ research include those by Ainsworth and Long (2005), Leakey et al. (2009), McCarthy et al. (2010), Norby, Wullschleger, Gunderson, Johnson, and Ceulemans (1999), and Oren et al. (2001). A key conclusion from these works is that we should expect positive vegetation responses to elevated atmospheric CO_2_ levels unless limited by environmental constraints.

#### 2.1.2 Warming

Hollister and Webber (2000) reported on the use and utility of the International Tundra Experiment—ITEX open‐topped passive warming chambers and established them as an appropriate analog for low levels of regional climate warming in tundra areas. While current projections of arctic warming now exceed the warming capacity of the ITEX chambers, other methods may be taking their place. Lewin, McMahon, Ely, Serbin, and Rogers (2017) reported on a new zero‐power warming system useful at remote sites that allows for a warmer treatment averaging +2.6°C setting the stage for any number of warming manipulations in remote but important ecosystems. Hanson et al. (2017) detail the methods for continuous whole‐ecosystem warming across a broad temperature range (+0 to +9°C), but such approaches are energy and infrastructure intensive and cannot easily be deployed in all important ecosystems.

#### 2.1.3 Drought

Fisher et al. (2007) studied the response of an eastern Amazonia forest to 50% reductions of throughfall to understand how tropical systems might respond to warmer and drier climates. They found no limitation of transpiration throughout the two monitored dry seasons as measured by the sap flow technique under ambient conditions, but the manipulation led to large dry‐season declines in transpiration. Restrictions on transpiration in the dry season were interpreted (via modeling) as a limitation of soil‐to‐root water transport, driven by low soil water potential and high soil‐to‐root hydraulic resistance. Peñuelas et al. (2007) executed a field experiment across European sites to understand shrubland responses to warming and drought. They found the relationship between annual biomass accumulation and soil moisture to be not significant at the wettest sites, but positive at driest sites. Responses to warming were strongest at the wettest sites. They further concluded that extreme events could change a trend of increased productivity in response to warming in the cold sites.

#### 2.1.4 Ozone

Mills, Hayes, et al. (2011) reported an up‐to‐date interpretation of the effects of ambient ozone pollution on vegetation involving both ozone‐sensitive and ozone‐resistant species and mapped vegetation response across Europe. Such data combining the results of experimental work and field observations provide the basis for the active management of tropospheric ozone pollution. Ainsworth, Yendrek, Sitch, Collins, and Emberson (2012) put such work in a global context and discuss implications for responses under climate change.

#### 2.1.5 Ocean acidification

Martin and Gattuso (2009) studied the effects of elevated partial pressure of CO_2_ and temperature, both alone and in combination, on crustose coralline algae in aquaria. The death of algae was observed only with elevated temperature and was higher under elevated *p*CO_2_. Associated with this death, net calcification decreased by 50% when both temperature and *p*CO_2_ were elevated, while no effect was found under elevated temperature and elevated *p*CO_2_ by themselves. Such results have major consequences for biodiversity and biogeochemistry in coralligenous communities. Kroeker et al. (2013) published a pivotal article on ocean acidification as a threat to marine species in the form of a meta‐analysis. The results demonstrated responses ranging from decreased survival, calcification, and changes in growth, development, and abundance in response to acidification when averaged across all organisms, but appropriately pointed out that the magnitude of these responses did vary among taxonomic groups. The authors concluded that acidification responses might be enhanced by elevated seawater temperature.

### 2.2 Experiments to reveal specific processes

While there is much overlap between experiments we define as “novel environment experiments” and “experiments to reveal specific processes,” our aim in this section is to highlight studies that have a specific focus on understanding the operation of a given process. The previous section focuses primarily on system‐level responses to environment while this section focuses on the mechanics of specific processes. Of course this is a spectrum rather than a dichotomy and perhaps the distinction is one of scale; nevertheless, we hope these two sides of the same coin are useful when considering experiments in ecology.

#### 2.2.1 Methane emission

Joabsson and Christensen (2001) established a relationship between rates of wetland plant production and CH_4_ emissions. They showed quantitatively that CH_4_ emissions were sensitive to net ecosystem exchange of CO_2_ and carbon turnover, concluded that the correlation resulted from vascular plant‐derived labile carbon, and tied the methane production activities to root system development. Kruger, Eller, Conrad, and Frenzel (2002), using stable isotopes in a flooded rice field, went on to further delineate multiple pathways for methane production and oxidation.

#### 2.2.2 Biodiversity loss in grasslands

Stevens, Dise, Gowing, and Mountford (2006) provided evidence that there had been a significant decline in the species richness and the cover of forbs throughout grasslands of Great Britain and correlated the loss of vegetation diversity with a gradient of nitrogen deposition. They found that the cause was due to grasses outcompeting other forms of vegetation because they took greater advantage of the nitrogen additions.

#### 2.2.3 Physiological acclimation

Kirschbaum (2004) used a model to interpret experimental work and shows that an apparent soil respiration acclimation to warming may instead be explained by a reduction in substrate, and Ellsworth et al. (2004) analyzed field CO_2_ response curves of 16 C3 species from a pine and deciduous forest, a grassland and a desert, and found species‐specific responses that were moderated by changes in leaf nitrogen.

#### 2.2.4 Range shifts of mobile populations

The rate of anthropogenic climate change is hypothesized to outpace the ability of organisms to reestablish themselves in more hospitable environments. While this seems to be a foregone conclusion for long‐lived terrestrial plant species, it may not be so for mobile populations of animals or certain life stages of aquatic organisms. In a recent *GCB* article, Crickenberger and Wethey (2018) discuss the nature of coastal populations of barnacles and how they may be impacted by warming trends that might expand northern boundaries and contract southern boundaries in the northern hemisphere. We are curious to read future studies for other species to determine if lost midlatitude niches might be replaced by new poleward niches, or if there are critical reproductive steps compromised by the accelerated nature of global climate change.

### 2.3 Network‐based studies across time or space

#### 2.3.1 Long‐term observations

While long‐term observations are not strictly experimental in nature, if they are executed over sufficient time and cover a broad range of natural variability for target variables they can reveal insights similar to those gained from experimental manipulations. For example, Lindroth, Grelle, and Moren (1998) reported on what were then early “long‐term” (two full years) eddy covariance observations of a boreal forest to show it to be a net source of carbon. They further interpreted the change in annual carbon balance to be highly sensitive to changing temperatures. This finding that a closed‐canopy forest could be a source of carbon over an extended period has changed our understanding of forests as carbon sinks. A similar example for drought response in temperature forests published elsewhere (Gu, Pallardy, Hosman, & Sun, 2016) reported on how multiyear eddy covariance and forest survey responses were able to reveal the level of precipitation reductions and associated changes in soil water availability necessary to drive tree mortality. Such results have not often been revealed from shorter term manipulation studies.

#### 2.3.2 Collaborative networks

Harmon et al. (2009) describe a long‐term litter decomposition study (LIDET) of decomposition processes across 27 sites. They developed regression equations for these sites that suggest that while a slow phase (0.139–0.221 year^−1^) is common among sites, it is not universal. The collective results indicated that the global store of litter estimated using only short term (i.e., faster decomposition rates) would be underestimated by at least one‐third.

### 2.4 Model‐experiment synthesis

Early comparative analyses of models to experiments set out largely to judge the efficacy of model projections or comparability of projections across models (Amthor et al., 2001; Hanson et al., 2004). In recent years, such efforts have expanded to include in‐depth analyses of the underlying mechanisms. Two recent notable publications in *GCB* have led such efforts. De Kauwe et al. (2013) diagnosed the primary causes of variability across 11 ecosystem models in their predictions of the response of forest water use and water use efficiency to experimental elevated CO_2_ treatments of eastern forests of the United States. The activity centered on the variable representations of process (mechanistic hypotheses and assumptions) used in the models. They revealed that differences, even subtle ones, or unexpected feedbacks from other assumptions in the stomatal response to elevated CO_2_ that couples carbon and water cycles had a large influence on predicted CO_2_ responses of forest water use and water use efficiency. Boundary layer coupling, canopy interception, and water stress were also identified as key processes in which alternative model representations had a substantial impact. Medlyn et al. (2016) took a different tack and used models as system‐level, mechanistic hypotheses for a priori predictions to help guide elevated CO_2_ experiments on Eucalypt woodlands. Such predictions have the potential to make more efficient use of money and time in the operation of large experiments.

## 3 FUTURE EXPERIMENTS

Understanding the responses of ecosystems to changes in climate and greenhouse gas concentrations will provide information to help us develop a sustainable relationship with our home planet. Therefore, ecological experiments will continue to be societally relevant. In order to maximize sustainability outcomes, social science should also be integrated into ecosystem experiment research (Mooney, Duraiappah, & Larigauderie, 2013), though we do not consider these aspects here. The interaction of warming temperatures, rising CO_2_, and altered precipitation may have nonlinear or threshold effects on key ecosystem properties and services such as vegetation survival, CO_2_ sequestration, and water production, yet the timing, magnitude, and location of such impacts cannot now be estimated with confidence due to lack of quantitative experimentation on the process understanding needed for such predictions. In this section, we advocate a hypothesis‐model‐experiment approach, champion the cause for a number of novel‐environment manipulations, discuss the importance of context in the interpretation of experiments, reiterate the utility of process studies, provide thoughts on the location of future studies, and comment on the cost of experiments and the need for community engagement.

### 3.1 Hypothesis‐model‐experiment synthesis

**Figure 2:**
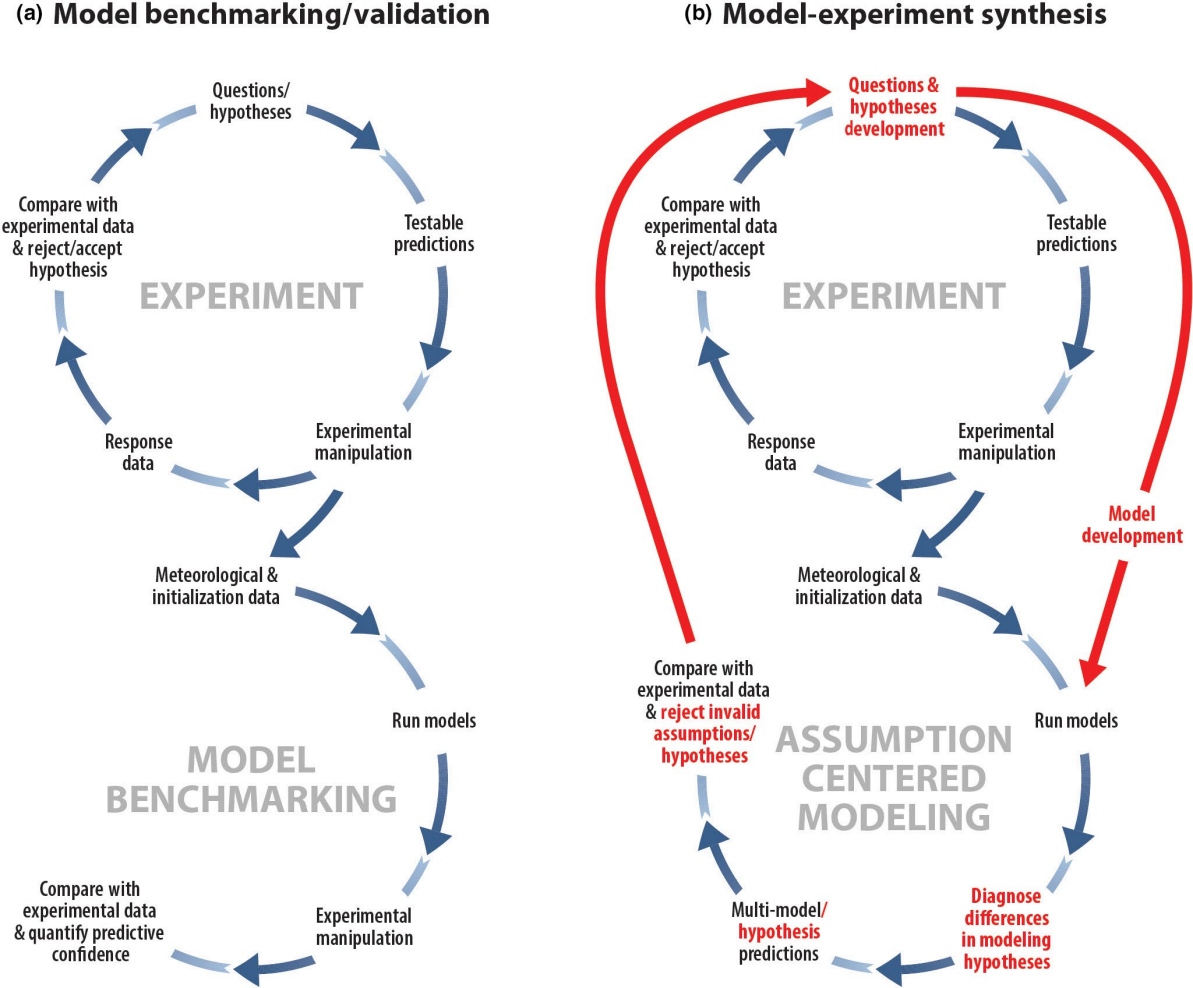
Diagram that compares traditional model benchmarking (a) and the assumption centered approach championed by the FACE‐MDS project (b) (from Medlyn et al., 2015; Walker et al., 2014). The assumption centered method goes beyond statistical evaluation of models' goodness‐of‐fit and diagnoses the behavior of the models in the context of their underlying process assumptions and hypotheses. The diagnosis describes model behavior in the language of science—mechanistic hypotheses and assumptions—and reconnects model results with experiment scientists. The integration of the assumption centered method into more traditional benchmarking brings models into the scientific method and is the core of the DOE ModEx philosophy (U.S. DOE, 2018)

Experiments to address specific science questions serve to test or discriminate among competing hypotheses that are alternative descriptions of how a biological or ecological process operates. Hypothesis, prediction, and evaluation of a prediction with experiment(s) are the key component of the scientific method. We illustrated above how ecosystem experiments fall into two broad categories—simulation of new environments or investigations of specific processes. Ecosystems are composed of a multitude of processes whose complex interactions result in the response of a given ecosystem to a given environmental change. Therefore, experiments that simulate a novel environment are investigating a suite of interacting processes, each of which is likely to have many associated hypotheses to be tested. Process‐based, simulation models are an integrated, system‐level hypothesis composed of hypotheses for key component processes of an ecosystem (Walker et al., 2018). Thus, ecosystem models are both predictive tools and quantitative, integrated hypotheses on the key mechanisms of an ecosystem (Figure 2).

Rastetter (2017) succinctly described the two sides of the modeling coin as “modeling for numbers” and “modeling for understanding,” and this dichotomy has been especially present in terrestrial ecosystem models due to the initial goals of predicting the land surface boundary condition in General Circulation Models (Pitman, 2003). As the complexity of ecosystem models grew, it became apparent that these models were more than predictive tools and should be integrated into the scientific method as system‐level hypotheses (Hanson et al., 2008; Medlyn et al., 2015; U.S. DOE, 2018; Walker et al., 2014).

Recent examples of post‐study, model‐experiment synthesis can be found in the work of the free‐air CO_2_ research community (e.g., De Kauwe et al., 2013; Medlyn et al., 2015; Zaehle et al., 2014). However, the experiments upon which these modeling studies are based were generally not designed with the aid of models. On the other hand, models are very often not designed with full support of experiments in mind (i.e., complete documentation of all the mechanistic hypotheses and assumption of which they are composed). This isolation of model and experiment slowed efforts at post hoc synthesis and made fully mechanistic explanations of observed responses difficult. Future observations and manipulations should be coupled with ecosystem process models at the very inception of a project. These models should be used to generate quantitative, system‐level predictions from the hypotheses of which they are composed. Modelers must provide a mechanistic interpretation of model results to identify process hypotheses and parameters that are responsible for model behavior, to guide measurement plans and, once data start to be collected, to interpret these results.

In support of this activity, modelers must make efforts to fully describe the process‐level scientific understanding (hypotheses and assumptions) of which their models are composed, maintain this information as models develop, and keep a clear record of what hypotheses (where alternatives exist) and parameter/trait values were used for a particular simulation. It is fine for models to be modified or calibrated to a certain system so long as a clear record of what was modified is maintained. Accurate descriptions from modelers of why their model behaves in a specific manner must become the norm.

### 3.2 Directions for future novel‐environment manipulations

**Figure 3:**
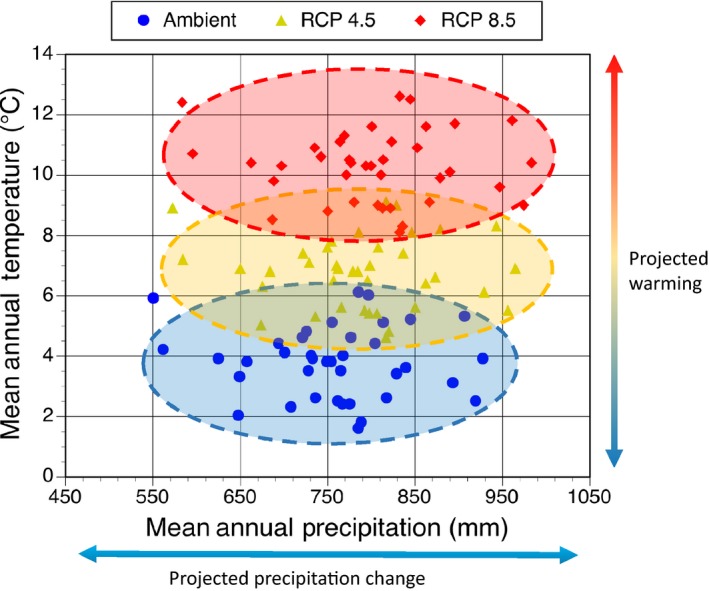
An example of historical and projected climate space (mean annual precipitation vs. mean annual temperatures) for a 50 year record in the eastern United States showing limited overlap between the known temperature record and projected temperature futures under a range of forcing scenarios. The temperature and precipitation projections are based on the model‐mean differential from IPCC (IPCC, 2013: Annex I) for RCP 4.5 and RCP 8.5 for the eastern United States region

There is no crystal ball to reveal the direction of future environmental research or the best possible experiments to understand how ecosystems may respond to environmental change. However, experiments that provide quantitative information for environmental scenarios that take us beyond historical analogs might be suggested as a priority (see also the editorial discussion in *GCB*: De Boeck et al., 2019; Korell, Auge, Chase, Harpole, & Knight, 2019). Figure 3 demonstrates with recorded temperature and precipitation data that scenarios of future warming in intact ecosystems take us beyond the record of past conditions, and essentially demand that manipulative studies be done to generate observations that cannot be obtained currently. A similar and perhaps stronger case can be made for elevated atmospheric CO_2_.

Our emphasis on warming and elevated CO_2_ is not to be interpreted as a conclusion that other environmental factors should not be pursued as main effect variables in an experimental context (such as precipitation or nutrient manipulation), but simply that projected levels of warming and CO_2_ atmosphere represent novel environments for every ecosystem and biome on the Earth. Historical records for precipitation (see also Figure 3) often include a wide range of precipitation conditions and variation through time covering both very wet and very dry conditions (if the record is sufficiently long). Precipitation variation shows that we might expect to gather appropriate water availability and drought data from long‐term observational records (e.g., Gu, Pallardy, Hosman, & Sun, 2015, 2016; Wood, Knapp, Muzika, Stambaugh, & Gu, 2018). However, if extremes become more extreme or more frequent, using projections of the mean change may not be the best statistic on which to base these conclusions. More severe or more frequent precipitation extremes may change the disturbance regime of a given ecosystem, as may changes in the magnitude or frequency of temperature extremes.

Figure 1 shows a trend in disturbance‐related experiments reported in *GCB* publications, from zero in the initial years of *GCB* to close to 30 in the most recent period. The rise in disturbance‐related experiments is encouraging as disturbance regimes are shifting with global change (IPCC, 2014) and new modeling methods are coming online to simulate disturbance and its consequences with more realism (e.g., Fisher et al., 2018; McCabe & Dietze, 2019). Changing disturbance regimes can reduce the resilience of ecosystems (e.g., Turner, Braziunas, Hansen, & Harvey, 2019). Disturbance resets the successional clock and can clear the way for previously uncompetitive species to get a foothold in a novel environment, potentially altering the response to global change compared with the previously established ecosystem. In addition, disturbance adds carbon to litter and soil pools and recovery from disturbance has different dynamics of resource limitation that could interact with rising CO_2_ (Andersen‐Teixeira et al., 2013; Rastetter et al., 2013). Experiments that monitor responses following a disturbance or manipulate an agent of disturbance are also likely to yield novel and interesting data to help improve our understanding of ecosystem responses to global change.

There have been a number of publications recommending the use of regression‐style approaches to more fully characterize response surfaces for variables measured within manipulative environmental changes studies (Cottingham, Lennon, & Brown, 2005; Fraser et al., 2013; Kayler et al., 2015). We endorse such approaches and have applied it to warming studies of peatland ecosystems (Hanson et al., 2017). Kreyling et al. (2018) make the case for using natural gradients or expanded gradients for manipulations to characterize nonlinear experimental response functions, but Chalcraft (2019) emphasizes the importance of replication.

### 3.3 Context dependence of experiment results

A key feature of ecological research is context dependence (e.g., Blonder et al., 2018; Kroeker, Kordas, & Harley, 2017; Smith‐Ramesh & Reynolds, 2017) and the results from manipulation experiments in ecology are no different. For example, Walker et al. (2019) emphasized that the observed progressive nitrogen limitation (PNL) of the net primary production response to CO_2_ observed at the ORNL FACE experiment was best understood in the context of PNL of ambient treatment production. In a study of 89 drought experiments, Hoover, Wilcox, and Young (2018) concluded that background precipitation variability was likely a key factor influencing variability results from the various drought experiments and that such variability must be considered when designing new experiments. Across 16 experiments that manipulated different environmental variables, Langley et al. (2018) showed that trends in species abundance over time in ambient treatments were often larger than the responses to the manipulation. Ignoring, or not evaluating treatment responses, in the context of these ambient treatment dynamics is likely to lead to erroneous conclusions. As a result, when context dependence is ignored it may be seen as weakness in ecological research. Indeed, understanding the context dependence of responses to environmental manipulation provides the strength and understanding from which the observed responses can be generalized.

Therefore, to explain ecosystem responses in complex environments and to aid model‐experiment synthesis, the research community must consider environmental variables beyond those manipulated. Ettinger et al. (2019) evaluated a range of warming studies and concluded that simplified analytical assessments of target warming treatments versus controls (e.g., ANOVA assessments of mean responses across replicates) instead of specific analyses of responses to imposed treatments (i.e., measured responses vs. the inherent variable nature of the imposed temperature treatments) were a lost opportunity to understand the complete nature of temperature response. They concluded that associated changes in other key variables (e.g., drying associated with warming) must be evaluated along with temperature change.

Experimental studies must allocate sufficient funding and effort to adequately measure not only the primary variables targeted for manipulation but also associated secondary variables known to have an influence on response measurements. A study focusing on experimental manipulations of temperature and elevated CO_2_, for example, must include adequate assessments of water and nutrient availability (e.g., Iversen, Hooker, Classen, & Norby, 2011). Future experimental work may also benefit further from the inclusion of associated characterization of atmospheric nutrient deposition and perhaps even routine monitoring of tropospheric ozone (Mills, Pleijel et al., 2011). When funding to study specific environmental variables changes with time (e.g., the transient attention to tropospheric ozone), the community should not forget the importance of previously emphasized variables to ecosystem processes or their contribution to dynamic biogeochemical cycles, as well as their importance for use as model driving data or model validation. Most would agree that such an approach is appropriate, but that it comes at a cost for investments in additional monitoring that may not have always been anticipated.

### 3.4 Process studies

While experiments to resolve primary environmental responses dominate *GCB* experimental publications, experimentation to better resolve process‐level understanding of key organism and ecosystem functions also advance global change science. Such experiments can be designed to define or reveal the mechanisms of specific processes to further our scientific understanding (e.g., Oberle et al., 2019). The tested mechanisms should be rigorously defined as mathematical hypotheses that generate quantitative predictions and that can be integrated with system‐level models.

While established and detailed mechanistic models to represent photosynthesis, stomatal function, and energy balance are available (e.g., Collatz, Ball, Grivet, & Berry, 1991; Collatz, Ribas‐Carbo, & Berry, 1992; Dubois, Fiscus, Booker, Flowers, & Reid, 2007; Farquhar, von Caemmerer, & Berry, 2001; Leuning, Kelliher, Depury, & Schulze, 1995), other processes are represented in less detail often with conceptual or empirical assumptions. In particular, processes controlling carbon storage and allocation are often represented with little detail even though this is well recognized and they have been studied extensively (Epron, Nouvellon, & Ryan, 2012). Advancements in model representations of dark respiration by plant tissues (Davidson, Samanta, Caramori, & Savage, 2012) and microorganisms (Wang et al., 2014) have been made in recent years, but further experimentation to test these models across a range of temperatures, elevated CO_2_ atmospheres, water availabilities, and across nutrient gradients would seem appropriate as a validation exercise for their function within ecosystem and Earth System models (e.g., Atkin, Bruhn, Hurry, & Tjoelker, 2005; Reich et al., 2008). Given the relatively long residence time of wood in an ecosystem, we argue that detailed model and experiment studies to illuminate the process of wood production, mortality, and decay are a high priority in carbon cycle science (Friend et al., 2019; Walker et al., 2019). Root functions of water transport and nutrient uptake are also critical processes for which some model frameworks exist and processes are understood, but the representation of such mechanisms within higher level models is also limited due to limited quantitative data on root traits and trait variation (Warren et al., 2015). Recent fine‐root trait databases will help to this end (e.g., Iversen et al., 2017) and it is likely that an understanding of how root and mycorrhizal fungi interact will be needed for mechanistic understanding of below‐ground ecosystem function (McCormack & Iversen, 2019).

### 3.5 Where should new experiments be conducted?

Future experiments should be judiciously deployed in highly sensitive, but globally relevant, ecosystems. Future experiments placed within Earth's biomes that are judged by model uncertainty assessments and research community agreement as key levers in the Earth System or highly sensitive to projected environmental changes will provide maximum benefit. These ecosystems are likely to be relatively under‐studied, with large global feedbacks, or subject to the largest environmental changes. Ecosystems sited in ecotones may yield useful information on how biomes may shift, and are likely to be subject to environmental variation, on top of any manipulation, that could yield useful insight into the mechanisms guiding range shifts, expansion, or contraction. Tropical rainforests, high‐latitude systems, dry shrublands and savannahs, coastal ecosystems, and coral reefs are high‐priority biomes for novel environment manipulations.

Process studies could, and perhaps should, be initially sited in well‐characterized and well‐understood systems in which the wealth of existing information can support deeper process understanding. Siting these studies in places that are readily accessible will allow regular access and characterization of the focal process. Process knowledge gained from these experiments could then be tested for generality and applicability by using focused manipulations across sites characterized by relevant environmental gradients.

### 3.6 Reality check

The high cost of experimental efforts (for both personnel effort and the application and upkeep of experimental manipulations) must include an associated commitment to a wide range of measurements. Characterization of primary treatment variables and associated environmental factors should be executed at spatial and temporal scales relevant to the organisms being studied. For example, while microbial responses may be appropriately associated with temperature, water, nutrient, and perhaps oxygen levels in their local environment, plant biological response might better be associated with a range of above‐ and belowground temperatures, and water and nutrient availability with the rooted soil profile.

Experimental measurement investments should not be short‐changed. All experimental units should be instrumented and should include more than single location assessments of key variables to cover vertical and horizontal variation within experimental plots. This is especially true for studies evaluating multiple types of organisms (e.g., trees, shrubs, forbs, moss, and microbes). Experimental studies must allocate sufficient funding and effort to adequately measure not only the primary variables targeted for manipulation but also associated secondary variables known to have an influence on response measurements.

Data collection should include a concomitant investment in the execution of a data management plan that leads to the long‐term retention of all relevant environmental and response variables. Such archives will be most useful and used if they are posted in public repositories without restrictions on their use (e.g., ESS‐DIVE, Dryad, or Zenodo, to name but a few). Data archival efforts are an efficient use of resources that provide an opportunity for analyses unforeseen by the original authors to be executed and add value to the initial investments.

These suggested enhancements or mandates for future experimental work have a very real cost in both time and financial resources. If implemented and embraced by the research community, they will produce better products for broader application and interpretation. It is incumbent on funding organizations, journals, and researchers to make the adoption of such enhancements a reality. Funding organizations must recognize the time and expense associated with robust, useful, and long‐term data archival by supporting the efforts of community data repositories and providing researchers with funds and a mandate to archive data. Researchers must recognize that the vast majority of data are generated using public, tax‐payer funds and thus must be publicly available. Some journals are now requiring archival of datasets prior to publication, we support this and argue that data archival pre‐review will help to make ecological research more robust.

### 3.7 Community engagement

What experiments lead to new published work in *GCB* or other journals will depend ultimately on an iterative dance between the research community, policy makers, and the public. The research community may propose work that they determine best addresses the next most important scientific questions, but such questions can be acted on only with the financial support of funding organizations or government agencies. To that end, global change researchers must seek out a broad engagement with the public, policy makers, and social scientists to understand societal needs (Mooney et al., 2013), to justify the benefits of new experiments against the costs, and to build support for the most compelling and relevant science questions and next‐generation experiments.

## 4 SYNTHESIS

A full range of experimental approaches covering small to large spatial scales will continue to be justified as a source for mechanistic understanding of ecosystem responses to global change. Novel‐environment manipulation experiments operating at ecosystem spatial scales that encompass the organismal, edaphic, and environmental diversity of the target ecosystem and allow for longer term carbon and nutrient cycle feedbacks will enable a full range of questions to be addressed. These large‐scale manipulations also allow for multidisciplinary participation of the science community necessary to fully understand ecosystem function (Osmond et al., 2004). We prioritize CO_2_, warming, and changing disturbance regimes as key agents of novel future environments. These experiments are likely to require advanced and novel engineering solutions to achieve their goals.

Integration of these ecosystem‐scale manipulations with more focused process‐based manipulations would aid more complete understanding of ecosystem responses, for example, the fertilization experiment at ORNL and EucFACE to confirm nitrogen or phosphorus (respectively) limitation of the forest ecosystem (Ellsworth et al., 2017; Iversen et al., 2011). Targeting wood production, mortality and decomposition, root function, and plant–microbe–soil interaction would yield beneficial process insights that would both help to explain ecosystem scale responses to manipulation and include more mechanistic hypotheses for the processes in the system‐level hypotheses represented by ecosystem models. Integrating the two above mentioned styles of experiment studies with a network of fused process studies or observations at well‐characterized sites, for example, NEON (Kao et al., 2012) or FLUXNET (Baldocchi et al., 2001) sites, and with spatially complete remote sensing products (Shiklomanov et al., 2019) would help to understand the mechanism and context dependence of the responses to allow accurate and robust generalization from costly, ecosystem scale manipulations. A nice example of a project that integrates ecosystem scale studies with both finer scale process studies and larger scale extensive studies is the Analysis and Experimentation of Ecosystems (AnaEE) France project (Clobert et al., 2018).

Any experiment should be integrated with quantitative, mechanistic modeling from the beginning, but the case for model‐experiment integration is even greater for integrated system‐level studies that aims for complete mechanistic understanding of ecosystem responses. Due to the complexity of ecosystem processes and the large range of scales relevant to global change biology (genes to the planet, seconds to centuries), full model‐experiment integration requires not just a single model, but a suite of models that cover multiple process hypotheses and multiple scales. Advanced modeling tools are coming online to represent multiple alternative hypotheses that will help speed hypothesis evaluation in a system's context and alternative model comparison (e.g., Sierra & Müller, 2015; Walker et al., 2018). However, true model‐experiment integration that can take advantage of all of our existing process knowledge and vast range of datasets will require a range of models and disciplinary expertise. To analyze existing data and to run and interpret the range of models necessary for informing new experiments in a timely manner, we advocate for the creation of a data synthesis and modeling center. A data synthesis and modeling center with the resources and skills that are required to understand and analyze the hierarchy of system's hypotheses that represent our mechanistic understanding of how ecosystems function within the Earth System.

## FUNDING INFORMATION

U.S. Department of Energy, Office of Science, Office of Biological and Environmental Research.
